# EIF4A3 Promotes Cell Proliferation via CDC5L Upregulation in Human Breast Cancer Cells

**DOI:** 10.7150/jca.108895

**Published:** 2025-03-03

**Authors:** Miao Zhang, Yuchen Xie, Shaoran Song, Ruiqi Wang, He Chen, Yazhao Li, Jie Liu, Juan Li, Yina Jiang, Peijun Liu, Bo Wang

**Affiliations:** 1 Center for Translational Medicine, the First Affiliated Hospital of Xi'an Jiaotong University, Xi'an 710061, Shaanxi, P.R. China.; 2 Key Laboratory for Tumor Precision Medicine of Shaanxi Province, the First Affiliated Hospital of Xi'an Jiaotong University, Xi'an 710061, Shaanxi, P.R. China.; 3 Department of Radiation Oncology, The First Affiliated Hospital of Xi'an Jiaotong University, Xi'an 710061, Shaanxi, P.R. China.; 4 Department of Pathology, The First Affiliated Hospital of Xi'an Jiaotong University, Xi'an 710061, Shaanxi, P.R. China.; 5 Department of Radiotherapy, Shaanxi Provincial People's Hospital, Xi'an 710068, Shaanxi, P.R. China.

**Keywords:** breast cancer, EIF4A3, CDC5L, cell proliferation

## Abstract

Breast cancer is one of the most common diseases affecting women's health. While research on breast cancer has made progress in recent years, it remains a major health concern. Studies have shown that the translation initiation factor EIF4A3 is closely related to the occurrence and development of tumors, but the specific mechanism is still unclear. In this study, we aimed to explore the specific molecular mechanism of EIF4A3 in promoting the malignant process of breast cancer *in vivo* and *in vitro*. Our results showed that the expression of EIF4A3 was significantly upregulated in breast cancer, and overexpression of EIF4A3 could accelerate the growth of breast cancer cells. RIP-seq and RIP-RT-qPCR analyses indicated that EIF4A3 can bind to the mRNA of CDC5L and influence its expression. From the catRAPID we predicted that EIF4A3-protein could bind to CDC5L by the 5705-5954 region of CDC5L-mRNA. CDC5L was the downstream effector of EIF4A3. These results suggested that the EIF4A3-CDC5L axis promotes the proliferation of breast cancer cells. This study provides a theoretical basis for understanding the role of EIF4A3 in the malignant process of breast cancer.

## Introduction

Breast cancer is the most common female cancer, with an estimated 297,790 new cases diagnosed in the United States in 2023^1^. Due to its complex origins, structural diversity, and regulation by hormones, there are still many challenges in treating breast cancer^2^. The mechanisms underlying the progression of breast cancer remain ill defined. Molecular targeted therapy can provide a new option for the treatment of cancers^3^. Therefore, it is urgent to find new evaluation indices and targets and to develop more effective treatments for breast cancer.

Eukaryotic translation initiation factor 4A isoform 3 (EIF4A3) belongs to the EIF4A family. The physiological effects of EIF4A family members are mainly affected by the RNA helicase activity stimulated by their binding proteins. Although EIF4A3 is highly homologous to EIF4A1 and EIF4A2, its helicase activity is not affected by its binding proteins EIF4B or EIF4H^4^. EIF4A3 can combine with MAGOH-Y14 and MLN51 to form the core component of the exon junction complex (EJC). Similar to other EIF4A family proteins, EIF4A3 can bind to single-stranded RNA (ssRNA) in an ATP-dependent manner^5^. The complex formed by the binding of EJC and mRNA plays a role at the nuclear pore, providing binding sites for other downstream effectors in the nonsense-mediated decay (NMD) process and transporting the spliced mRNA to different parts of the cell^6^. In fact, the role of EIF4A3 depends almost entirely on its inability to perform the classical function of helicase. Several studies have reported on the role of EIF4A3 in cancer. Four EIF4A3 binding sites are found in the upstream region of circMMP9 transcript mRNA, and EIF4A3 can regulate the expression of circMMP9. Overexpression of circMMP9 enhances the proliferation, invasion, and migration abilities of glioma cells^7^. In addition, EIF4A3 can induce the expression and nuclear output of circPRKCI in triple-negative breast cancer (TNBC) cells. CircPRKCI can promote the progression of TNBC by regulating the WBP2 and PI3K/AKT signaling pathways^8^. Taken together, EIF4A3 plays an important role in the occurrence and development of tumors and is an important molecule that regulates tumor growth. However, the specific regulatory mechanism is still unclear. We found that EIF4A3 could bind to the mRNA of CDC5L and regulate its expression.

Cell division cycle 5-like protein (CDC5L) is a key regulatory factor in the process of mitosis and is also an mRNA presplicing factor. Low expression of CDC5L can cause kinetochore microtubule attachment and severe DNA damage, leading to significant mitotic arrest, chromosome dislocation, and sustained activation of spindle assembly checkpoints^9^. Previous studies have shown that CDC5L is highly expressed in tumors and involved in the occurrence and development of colorectal cancer, bladder cancer, breast cancer and other tumors. Li J *et al.*^10^ found that knocking down CDC5L can downregulate the expression of hTERT and inhibit tumor growth, indicating that CDC5L may be a new therapeutic target for human colorectal cancer. In addition, CDC5L is highly expressed in bladder cancer, and its expression is significantly related to the pathological grade of bladder cancer and Ki67 expression^11^. In breast cancer, the phosphorylation inhibitor CVT-313 of CDC5L has high cytotoxicity in breast cancer cells^12^.

Overall, EIF4A3 and CDC5L are expected to become tumor biomarkers. However, the specific mechanism of the relationship between EIF4A3 and CDC5L in breast cancer is still unclear and requires further clarification. In the present study, we mainly focused on exploring the role and detailed mechanism of EIF4A3 in the proliferation of human breast cancer cells. Our results highlight a novel mechanism underlying the growth of breast cancer cells and suggest that the EIF4A3-CDC5L axis plays an important role in breast cancer progression and might be a potential therapeutic target for breast cancer.

## Materials and methods

### Cell lines and cell culture conditions

The MCF10A, MCF-7, MDA-MB-231, T47D and BT-549 cell lines were purchased from Shanghai Institute of Biochemistry and Cell Biology (National Collection of Authenticated Cell Cultures, Shanghai, China). Human breast cancer cell lines MCF-7 and MDA-MB-231 were cultured in high glucose Dulbecco's Modified Eagle's medium (HyClone) supplemented with 10% fetal bovine serum (FBS, HyClone). The human breast cancer cell lines T47D and BT-549 were cultured in Roswell Park Memorial Institute (RPMI)-1640 medium (HyClone) supplemented with 10% FBS and either 10 μg/ml or 1 μg/ml insulin (Sigma), respectively. MCF-10A cells were cultured in DMEM/F12 medium (HyClone) with all recommended supplements. All cells were maintained in a 37°C, 5% CO_2_ incubator for culture.

### Bioinformatic analyses

The survival plots and the box plots of expression of EIF4A3 and CDC5L in breast cancer were from the Breast Cancer Integrative Platform (BCIP), http://www.omicsnet.org/bcancer/database. Analysis of proteins interacting with EIF4A3 was performed in the Interacting Proteins for EIF4A3 Gene of GeneCards®: The Human Gene Database (https://www.genecards.org/), which linked with the String database (https://cn.string-db.org/). The catRAPID website (http://service.tartaglialab.com/page/catrapid_group) was used to predict the bind of EIF4A3-protein and CDC5L-mRNA.

### RNA interference, plasmid transfection and lentiviral infection

The corresponding siRNAs and plasmids were designed and synthesized by GenePharma Biotechnology (Shanghai, China). The sequence of siRNAs was shown in **Table S1**. Lipofectamine 2000 (Invitrogen, Carlsbad, CA, USA) was used for the transfection of siRNAs and plasmids. In brief, cells were inoculated into 6-wells plates so they were 70-90% confluent at the time of transfection. Then cells were transfected with the mixture of 4μg Plasmid and 9 μL Lipo 2000 or 75 pmol siRNA and 7.5 μL Lipo 2000. After 48h of transfection, cells were collected and the efficiency of transfection was determined.

Lentiviral shRNA-negative control (Lv-shNC) and shRNA-EIF4A3 (Lv-shEIF4A3) viruses were obtained from GeneChem (Shanghai, China) and constructed using the above siRNA sequences. MCF-7 cells (2× 10^5^) were inoculated into 6-wells plates, infected with lentivirus and taken MOI= 20 as the standard. The cells were treated with 1μg/ml puromycin after seventy-two hours of infection. Then the stable cells were obtained.

### RNA isolation and real-time RT‒qPCR

Total RNA was obtained using the RNAfast 200 kit (Fastagen Biotechnology, Shanghai, China), and cDNA was converted with PrimeScript RT Master Mix (Takara Biotechnology, Dalian, China). RT‒qPCR was performed with SYBR Green qPCR Mix (Yeasen Biotechnology, Shanghai, China) according to the manufacturer's instructions. The Bio-Rad CFX96TM Real-Time PCR Detection System was used for qPCR analyses. The expression of mRNA was normalized to GAPDH. The sequence of primer sets was shown in **Table S2**.

### RNA immunoprecipitation sequencing (RIP-seq)

RIP assays were performed in MCF-7 cells using the Magna RIP™ RNA-Binding Protein Immunoprecipitation Kit (Merck, Germany). Briefly, cells were lysed in lysis buffer after collection. Antibodies and target RNA-binding proteins were enriched by using A/G magnetic beads. The magnetic bead binding compound was fixed with a magnetic frame, and then the unbound materials were washed off with RIP washing buffer. RNA was obtained after extraction and purification, and concentration analysis was performed. Then, rRNAs were removed to retain mRNAs and ncRNAs. The enriched mRNAs and ncRNAs were fragmented into short fragments by using fragmentation buffer and reverse transcribed into cDNA with random primers. Second-strand cDNA was synthesized by DNA polymerase I, RNase H, dNTP (dUTP instead of dTTP) and buffer. Next, the cDNA fragments were purified with a QiaQuick PCR extraction kit, end repaired, poly(A) added, and ligated to Illumina sequencing adapters. Then, uracil-N-glycosylase (UNG) was used to digest the second-strand cDNA. The digested products were size selected by agarose gel electrophoresis, PCR amplified, and sequenced using Illumina HiSeqTM 4000 by Gene Denovo Biotechnology Co. (Guangzhou, China). To identify regions of IP enrichment over background, we used the RIPSeeker^13^ algorithm, which is a de novo method for detecting peaks (indicating protein-RNA interactions) based on hidden Markov models (HMM). eFDR < 0.05 and IP count > 10 were used to select peaks. According to the genomic location information and gene annotation information of peaks, peak-related genes can be confirmed. In addition, the distribution of peaks in different functional regions, such as protein_coding, pseudogene and antisense regions, was determined. MEME suite (http://meme-suite.org/) was used to detect the significant sequence motif analysis in the transcript sequence associated with peaks. For validation, RNA that was obtained from RIP was analysed using RT‒PCR.

### MTT assay

Cells were plated equally into a 48-well plate after collection and counting. At regular intervals of 1, 2, 3 and 4 days, cells were treated with 50 μL MTT (5 mg/mL) and incubated at 37°C for 4 h. When time was over, the medium was removed, and 375 μL of DMSO was added to dissolve the formazan crystals. The absorbance was determined at 490 nm using a microplate reader (PerkinElmer).

### Colony formation assay

Cells were seeded into 6-well plates and incubated at 37°C with 5% CO_2_ for 2 weeks. After discarding the old culture medium and washing with PBS twice, the cells were fixed with methanol for 15 mins and stained with crystal violet for 20 mins.

### EdU incorporation assay

A 96-well plate was used for the culture of cells. After seeding, the cells were incubated with 10 μM EdU for 2 hours. According to the kFluor555 Click-iT EdU detection kit (KeyGen Biotech, Nanjing, China), EdU detection was performed. Cells were imaged with a Leica DMi8 Microscope (Leica, Germany).

### Western blotting

Protein extraction was performed with RIPA lysis buffer containing protease and phosphatase inhibitors, and Bio-Rad protein assay reagent (Bio-Rad) was used to estimate the protein concentration. Cell extracts were separated by sodium dodecyl sulfate‒polyacrylamide gel electrophoresis (SDS‒PAGE) and transferred to polyvinylidene difluoride membranes (PVDF, Millipore). Subsequently, the membranes were blocked with 5% fat-free milk in TBST for an hour and then incubated with primary antibody overnight at 4°C. The next day, the membranes were cleaned and incubated with horseradish peroxidase-conjugated secondary antibody for an hour at room temperature (1:10000; Proteintech). Chemiluminescent signals were detected using electrochemiluminescence (Bio-Rad). Primary antibodies against EIF4A3, MCM7, p27 and CDC5L were purchased from Santa Cruz, and antibodies against Survivin, p21, CyclinD1, GAPDH and β-actin were obtained from Proteintech.

### Immunohistochemistry

The breast cancer specimens and adjacent normal tissues in this work were from the First Affiliated Hospital of Xi'an Jiaotong University. This study was permitted by the Ethics Review Committee of the First Affiliated Hospital of Xi'an Jiaotong University and informed consent has been obtained from the guardians of all patients. Clinical tissue samples were handled according to the ethical standards outlined in the Declaration of Helsinki.

Inclusion criteria:

a. Patients with breast cancer.

b. Aged between 18 and 65 years old.

c. Karnofsky Performance Status (KPS) score greater than 60.

d. The maximum diameter of the tumor is greater than or equal to 3 cm.

e. No prior use of any chemotherapy regimen.

f. Good functions of the heart, liver, kidneys and bone marrow hematopoiesis.

Exclusion criteria:

a. Pregnant or lactating women.

b. Patients with other concurrent tumors.

c. KPS score less than 60.

d. Occurrence of severe cardiovascular and cerebrovascular events within half a year, presence of diseases that have not been cured and affect prognosis; other important systemic diseases (such as severe infections and trauma, malnutrition, dehydration, major surgical operations, etc.).

e. Patients in a mental state where they cannot understand the nature, scope and possible consequences of this study, including evidence of an officially non - cooperative attitude and/or inability to accept follow - up, impossibility of completing the study or failure to comply with subsequent medical guidance, lack of the ability to complete the study, and poor compliance.

The tissues were fixed in 10% neutralized formaldehyde and sliced into 4-μm-thick paraffin sections. We used a Biotin-Streptavidin HRP Detection System (ZSGB-BIO, Beijing, China) to perform IHC staining. Images were taken by a Leica SCN400 slide scanner (Germany). The scoring method was as follows: ten random visual fields were selected for each tissue slice, and semiquantitative scoring was used for the estimation of tissue staining in each visual field. Positive cell rate integration method: if there were no positive cells or the proportion of positive cells was less than 10%, 0 points were counted; positive cells accounted for 10% to 25%, 1 point was counted; positive cells accounted for 25%~50%, 2 points; positive cells accounted for 50%~75%, 3 points; positive cell ratio>75%, 4 points. The dyeing intensity integration method was as follows: no staining of cells, 0 points; light yellow color, 1 point; brown yellow color, 2 points; brown color, 3 points. The result was determined by the product of the positive cell rate and staining intensity: a product of 0 indicated negative (-), and a product of 1-4 indicated weak positive (+); a product of 5-8 was recorded as positive (++); a product of 9-12 was recorded as strongly positive (++).

### Apoptosis assays

Treated cells were collected and stained with 7-AAD and Annexin V according to the apoptosis detection kit (BD Biosciences). The results were analyzed by flow cytometry (FCM, BD Biosciences).

### *In vivo* assay

Four-week-old female BALB/c nude mice were purchased from the Research Animal Centre of Xi'an Jiaotong University (Xi'an, China), and the control and experimental groups were established by random allocation (n = 5 per group). A total of 5× 10^6^ shRNA-negative control or shRNA-EIF4A3 MCF-7 cells were injected into the mammary fat pad of mice after resuspending in 100 μL PBS. The tumor size was measured every 3 days, and the tumor weight and volume were assessed after collection on the 28th day. The volume = (length × width^2^)/2. Images were taken, and the tumors were fixed to make 4-μm-thick paraffin sections.

### Statistical analysis

All experiments were repeated at least three times *in vitro*. The data were analyzed and processed using GraphPad Prism 9. Data are presented as the mean ± standard deviation (SD). Student's *t* test was used to compare the differences between the two groups for statistical significance. Two-way *ANOVA* was used to compare multiple sample averages. Statistical significance was considered when *P* < 0.05 (*P < 0.05, **P < 0.01, ***P < 0.001, and ****P < 0.0001).

## Results

### EIF4A3 is highly expressed in breast cancer

**Figure 1:**
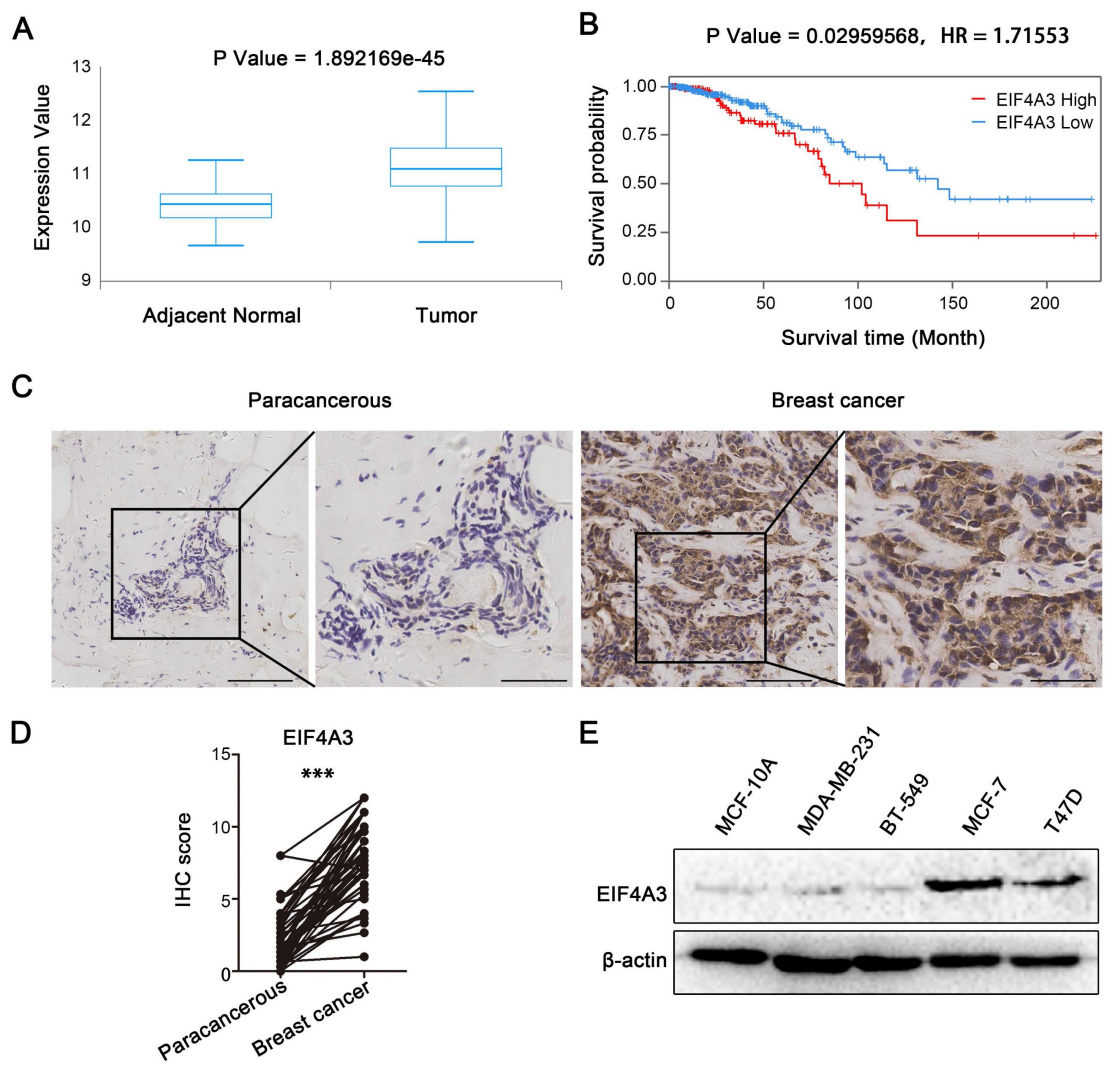
High-level expression of EIF4A3 is closely related to poor prognosis of patients with breast cancer. A: Expression of EIF4A3 mRNA in breast cancer and adjacent tissues in TCGA; B: Correlation between EIF4A3 mRNA expression level and overall survival (OS) of breast cancer patients in TCGA; C-D: Expression of EIF4A3 in breast cancer and adjacent tissues by immunohistochemistry method (Scale bar: 100 μm on the left and 50 μm on the right, ***P<0.001); E: Expression of EIF4A3 protein in immortalized breast epithelial cells and breast cancer cell lines by Western blotting.

We analyzed the correlation between EIF4A3 expression and the survival probability of breast cancer patients in the TCGA database to investigate the potential roles of EIF4A3 in breast cancer. The box plot showed the expression of EIF4A3 in adjacent normal and tumor tissues in TCGA (**Figure 1A**), from which we found that EIF4A3 had higher mRNA expression in tumor samples than in adjacent normal samples. Kaplan‒Meier plot analyses indicated that patients in the high EIF4A3 mRNA expression group had a shorter survival time and lower survival rate than those in the low expression group (**Figure 1B**). Next, we collected 47 pairs of human breast cancer tissues and 60 nonpaired human breast cancer tissues to explore the expression of EIF4A3 by utilizing immunohistochemical staining. The clinicopathological associations of EIF4A3 in human breast cancer were provided in **Table S3**. The results showed that EIF4A3 staining signals were much higher in breast cancer tissues than in adjacent tissues (**Figure 1C and D**). The protein levels of EIF4A3 in immortalized cells (MCF10A) and several breast cancer cells (MCF-7, T47D, BT-549 and MDA-MB-231) were shown in **Figure 1E**, which indicated that EIF4A3 expression was relatively high in MCF-7 and T47D cells. These results confirm that EIF4A3 was likely associated with the development of breast cancer.

### Knockdown of EIF4A3 suppresses proliferation and accelerates apoptosis in human breast cancer cells

**Figure 2:**
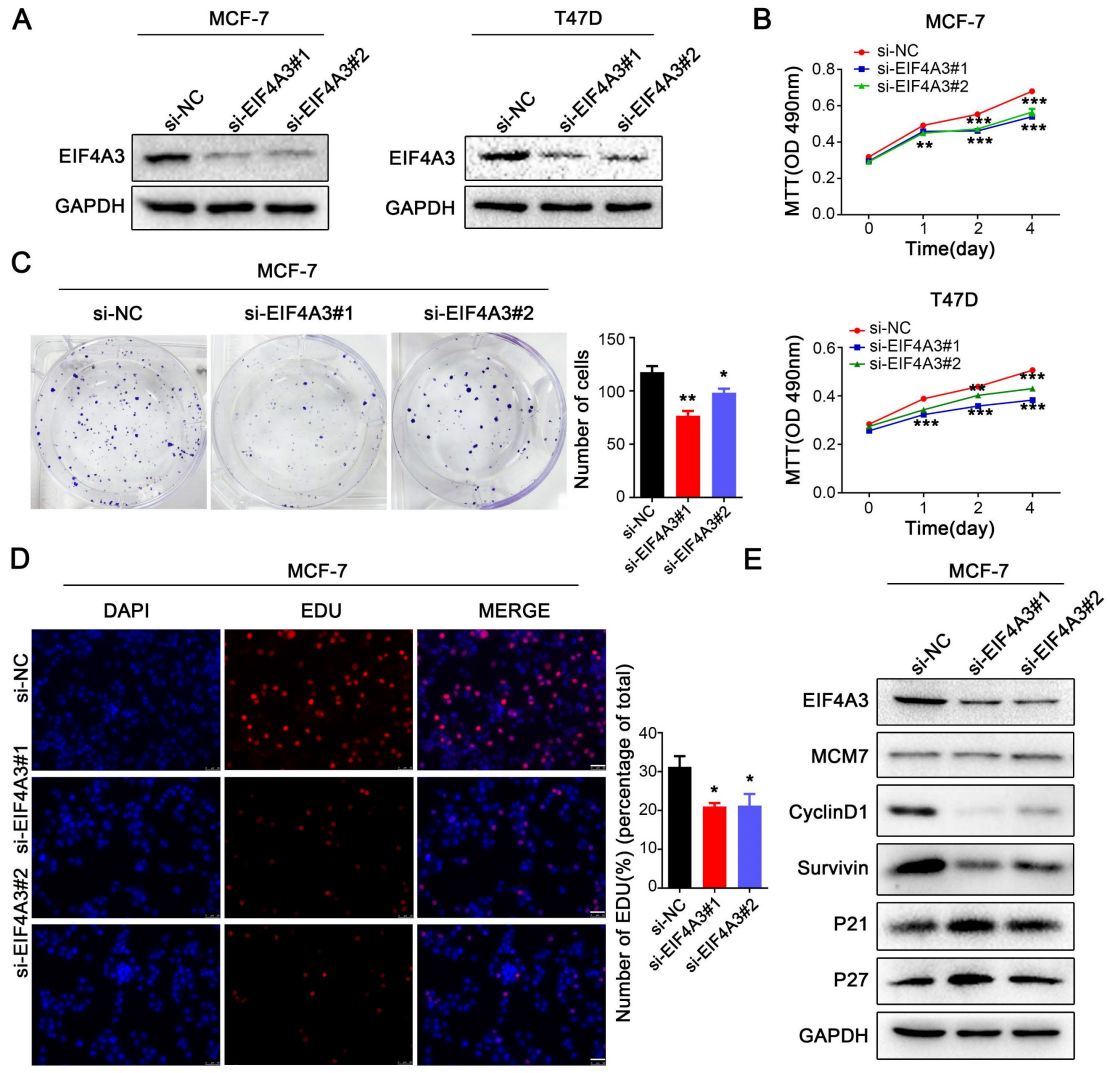
Knockdown of EIF4A3 suppresses the proliferation of breast cancer cells. A: The knockdown efficiency of EIF4A3 was detected by western blotting in MCF-7 and T47D cells; B-D: The effect of knocking down EIF4A3 on the proliferation of MCF-7 or T47D cells was detected by MTT (B), colony formation (C) and EdU incorporation (D, Scale bar: 50 μm) (*P<0.05, **P<0.01, ***P<0.001); E: Analysis of MCM7, CyclinD1, Survivin, P21 and P27 expression in MCF-7 cells by Western blotting.

To investigate the role of EIF4A3 in the biological functions of breast cancer cells, we used two siRNAs to knock down the expression of EIF4A3 in MCF-7 and T47D cells (**Figure 2A**). We performed MTT assays, colony formation assays, flow cytometric apoptosis analysis and EdU incorporation to determine the effect of EIF4A3 depletion on cell proliferation and the cell cycle. The MTT assay showed that cell proliferation was significantly inhibited in both MCF-7 and T47D cells (**Figure 2B**). The number of colonies formed was distinctly decreased in the EIF4A3-depleted group (**Figure 2C**). Flow cytometry analysis revealed that the ratio of apoptotic cells increased after the downregulation of EIF4A3 expression (**Figure S1A**). Additionally, a decrease in cells in the S-phase was observed by EdU incorporation assay in MCF-7 cells (**Figure 2D**). We also assessed the expression levels of a series of cell cycle regulators after the knockdown of EIF4A3 using western blotting. We observed no differences in the expression levels of MCM7 but found that EIF4A3 was markedly related to p27, p21, CyclinD1 and Survivin (**Figure 2E**). Taken together, our data suggested that the knockdown of EIF4A3 inhibits cell proliferation and promotes apoptosis in human breast cancer cells.

### EIF4A3 overexpression accelerates the proliferation of human breast cancer cells

**Figure 3:**
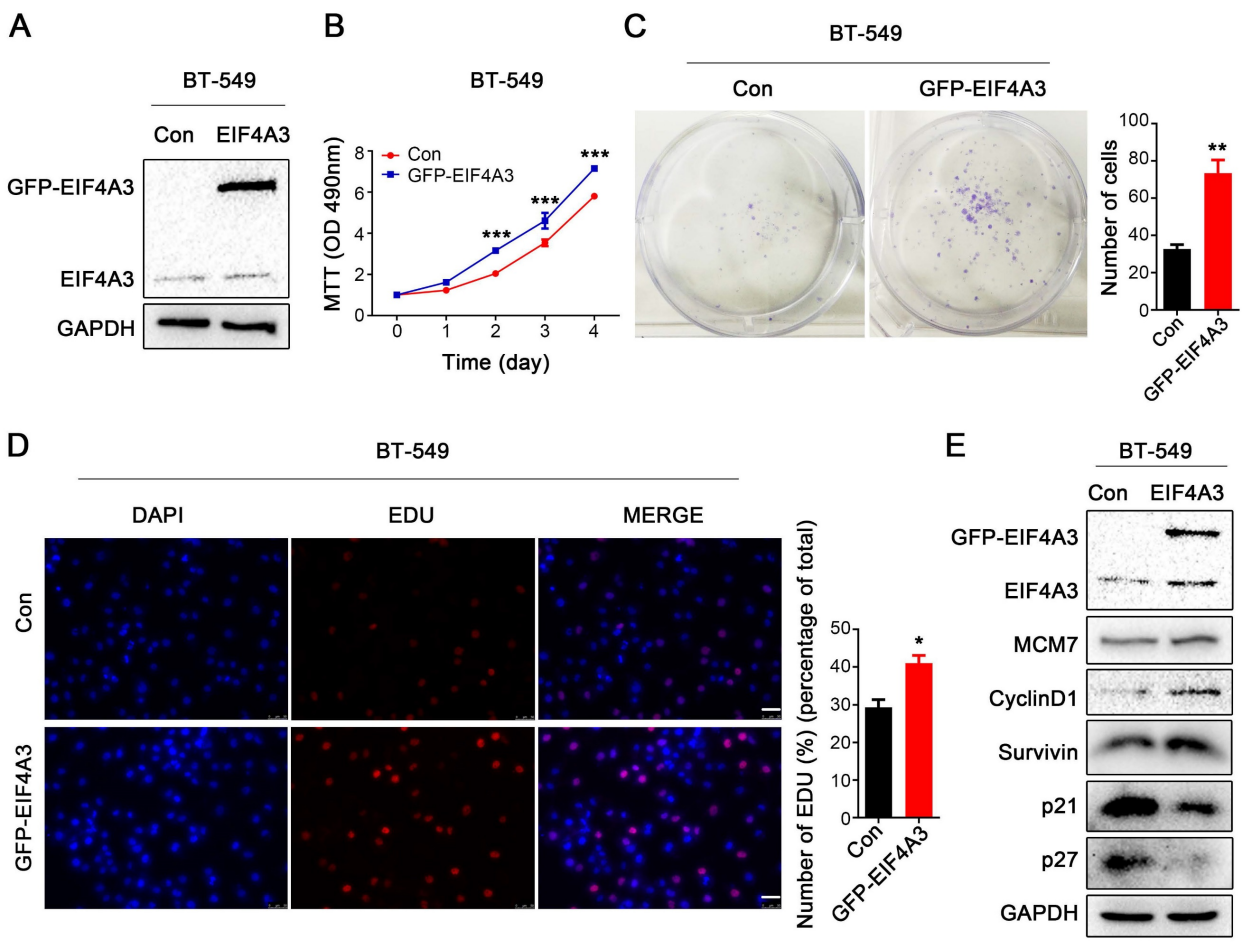
EIF4A3 overexpression accelerates the proliferation of breast cancer cells. A: The overexpression efficiency of EIF4A3 was detected by Western blotting in BT-549 cells; B-D: The effect of upregulation of EIF4A3 expression on the proliferation of BT-549 cells was detected by MTT (B), colony formation (C) and EdU incorporation (D, Scale bar: 50 μm) (*P<0.05, **P<0.01, ***P<0.001); E: Analysis of MCM7, CyclinD1, Survivin, P21 and P27 expression in BT-549 cells by Western blotting.

Subsequently, we overexpressed GFP-tagged EIF4A3 in BT-549 cells due to its low expression of EIF4A3 (**Figure 3A**). Notable increases in cell growth viability and colony formation were observed after EIF4A3 overexpression (**Figure 3B, C**). However, the flow cytometry assay showed no difference between the control group and the EIF4A3 overexpression group (**Figure S1B**). When EIF4A3 was overexpressed, the number of S-phase cells increased (**Figure 3D**). Although there was no obvious change in the expression of MCM7, EIF4A3 overexpression was positively correlated with the expression of Survivin and CyclinD1and negatively correlated with the expression of p21 and p27 (**Figure 3E**). These data indicated that EIF4A3 overexpression promotes cell proliferation in human breast cancer BT-549 cells.

### EIF4A3 promotes tumorigenic ability *in vivo*

**Figure 4:**
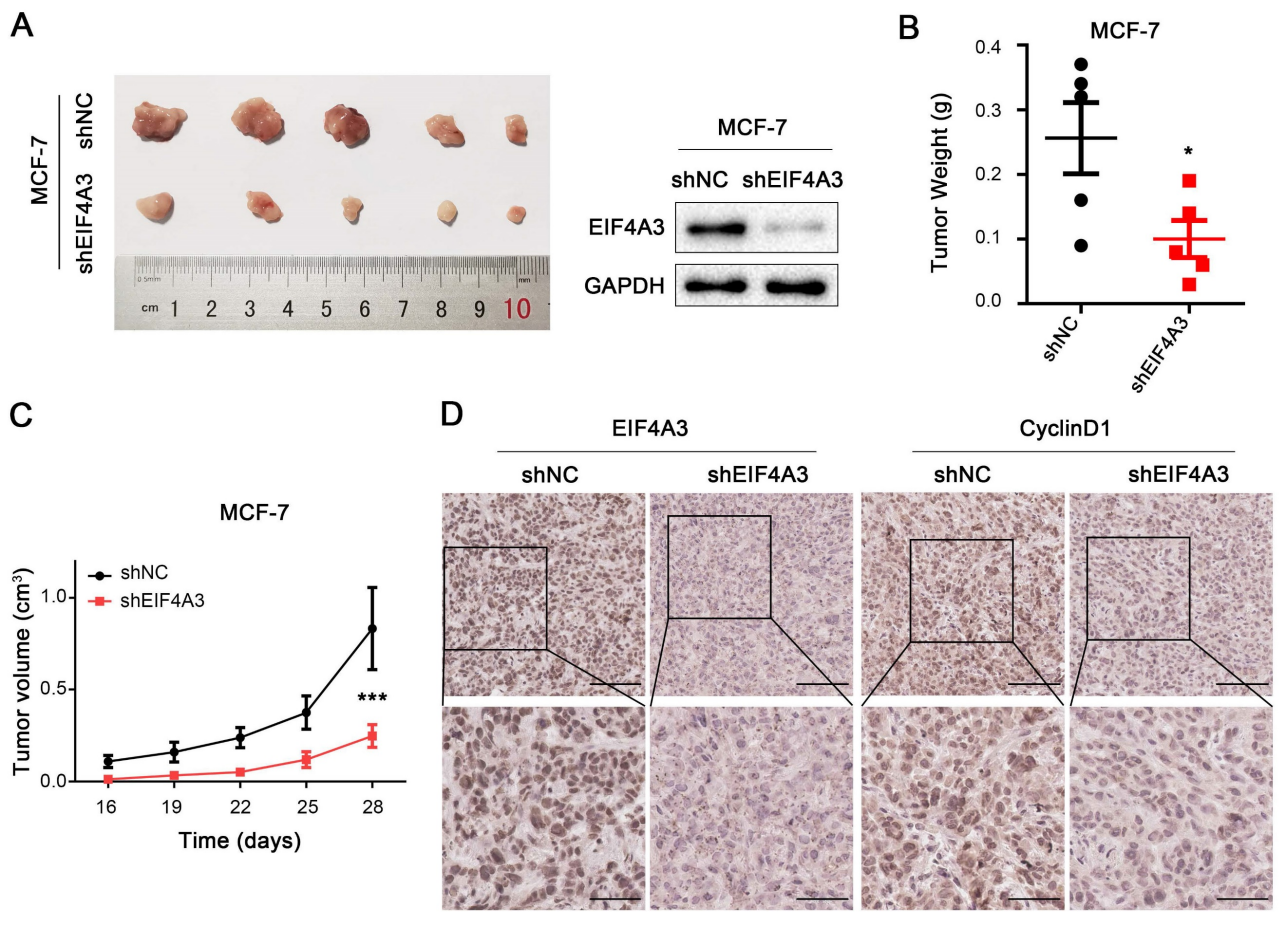
EIF4A3 promotes tumorigenic ability *in vivo*. A: EIF4A3 stably depleted and negative control MCF-7 cells were used to establish a mouse xenograft tumor model. B-C: Tumor weight (B) and volume (C) were quantified (*P<0.05, ***P<0.001). D: Analysis of EIF4A3 and CyclinD1 expression in xenograft tumors by immunohistochemistry (Scale bar: 100 μm on the top and 50 μm at the bottom).

To further investigate the effects of EIF4A3 on cell proliferation *in vivo*, we constructed a xenograft tumor model using stable sh-NC-MCF-7 and sh-EIF4A3-MCF-7 cells. Photos of the tumors after 4 weeks were taken and shown in **Figure 4A**, which confirmed the efficiency of EIF4A3 protein knockdown in sh-EIF4A3-MCF-7 cells. The tumor weight in the EIF4A3-depleted group was significantly lighter than that in the control group (**Figure 4B**), and although the tumors in both groups gradually increased in volume, the tumor volume in the control group were much larger (**Figure 4C**). Immunohistochemical staining showed that EIF4A3 and CyclinD1 were consistently lower in expression in the tumors of the EIF4A3-depletion group (**Figure 4D**). In conclusion, our findings demonstrated that knockdown of EIF4A3 inhibits breast cancer cell growth *in vivo*.

### Analysis of binding RNAs of EIF4A3 by RIP-seq

**Figure 5:**
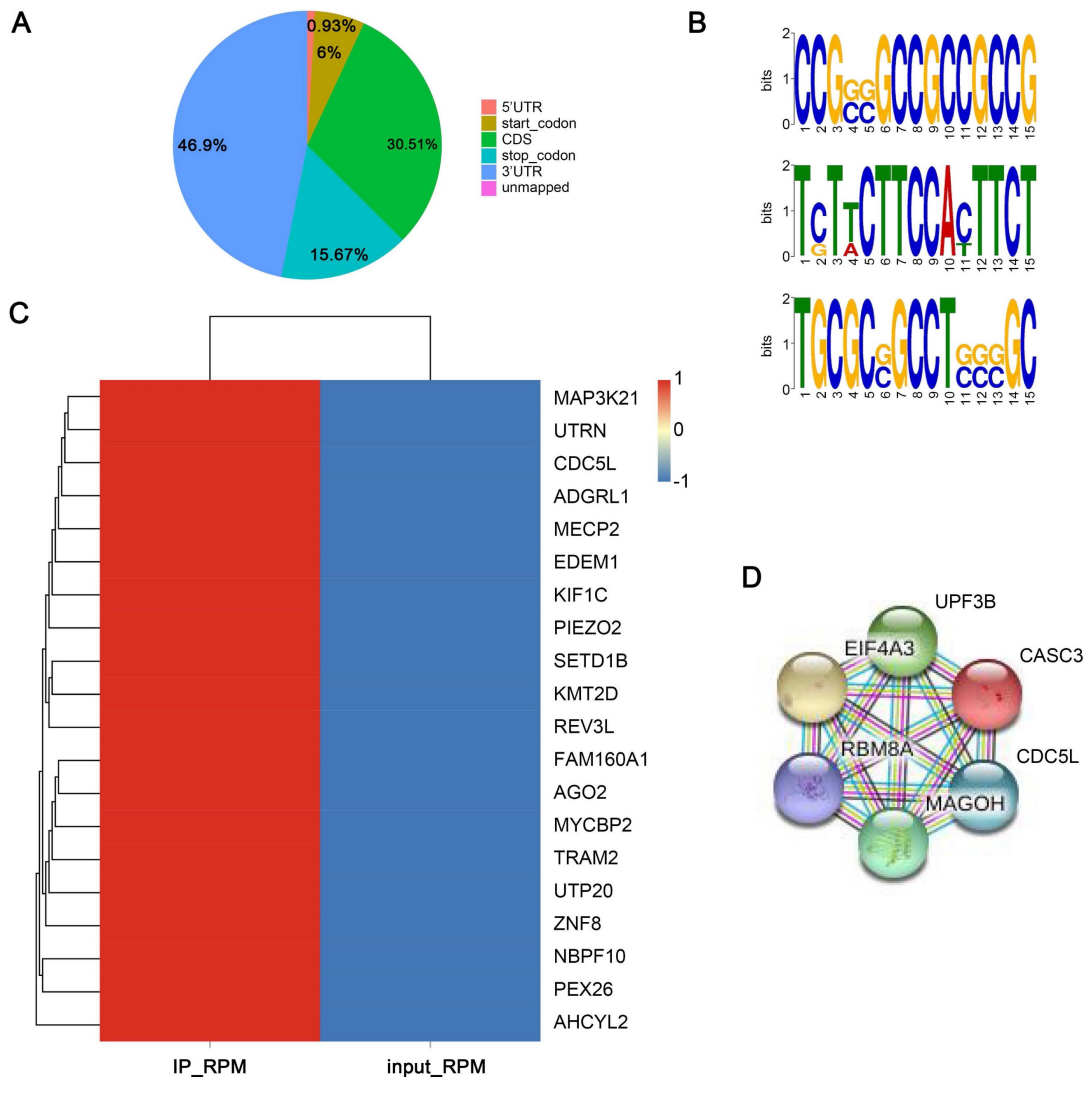
Analysis of binding RNAs of EIF4A3 by RIP-seq. A: Distribution and percentage of peaks on different functional elements of coding genes. B: Top three sequence motifs identified from MeRIP-seq peaks. C: The top 20 binding mRNAs of EIF4A3 from RIP-seq. D: Analysis of proteins interacting with EIF4A3 in the String database.

As an RNA binding protein, EIF4A3 can bind to and regulate numerous RNAs. To explore the binding RNAs of EIF4A3, we employed RIP-seq for further study. The results in **Figure 5A** showed the distribution of peaks in different functional regions, including the 5'UTR, start_codon, CDS, stop_codon and 3'UTR. We found that peaks were most enriched in the 3'UTR, with a percentage of 46.9%. Significant sequence motif analysis in the transcript sequence associated with peaks was performed to detect motif sequences and the preference of nucleic acid sequences of EIF4A3 protein-specific binding sites. The “GCC” sequence motif was verified to be highly enriched in EIF4A3-immunoprecipitated RNAs (**Figure 5B**) (Different colors represent different base types, and the height of the letter represents the conservation of the base. The higher the letter, the more frequently it appears throughout the site, and the more conserved it is.). The peak-related genes were divided into four types, including lncRNA, mRNA, nonsense-mediated decay, and processed transcript, with mRNAs being the focus of this study. The heatmap displayed the top 20 binding mRNAs of EIF4A3 (**Figure 5C**). The gene CDC5L came to a highlight with the common analysis of RIP-seq and interaction protein of EIF4A3 on the STRING database (**Figure 5D**), and there was no relevant report in the literature. Collectively, these findings implied that EIF4A3 might be very important for the expression of CDC5L.

Subsequently, the catRAPID website was used to predict the bind of EIF4A3-protein and CDC5L-mRNA. The interaction profile showed the score (y-axis) of protein-RNA interactions along RNA sequences (x-axis) (**Figure S2A**) and the interaction matrix displayed the predicted interactions between protein (y-axis) and RNA (x-axis) regions (**Figure S2B**). The depth of the red markings on the heatmap represents the interaction score between individual amino acids and nucleotide pairs. **Table S4** was the prediction results, in which including the protein region, the RNA region, the raw score (for graphical representation) and the discriminative power (DP, DP value above 50% indicates that an interaction may occur, while a DP value above 75% indicates a high confidence prediction.) of each interaction. From the catRAPID we predicted that EIF4A3-protein can bind to CDC5L by the 5705-5954 region of CDC5L-mRNA.

### CDC5L expression is closely linked to EIF4A3

**Figure 6:**
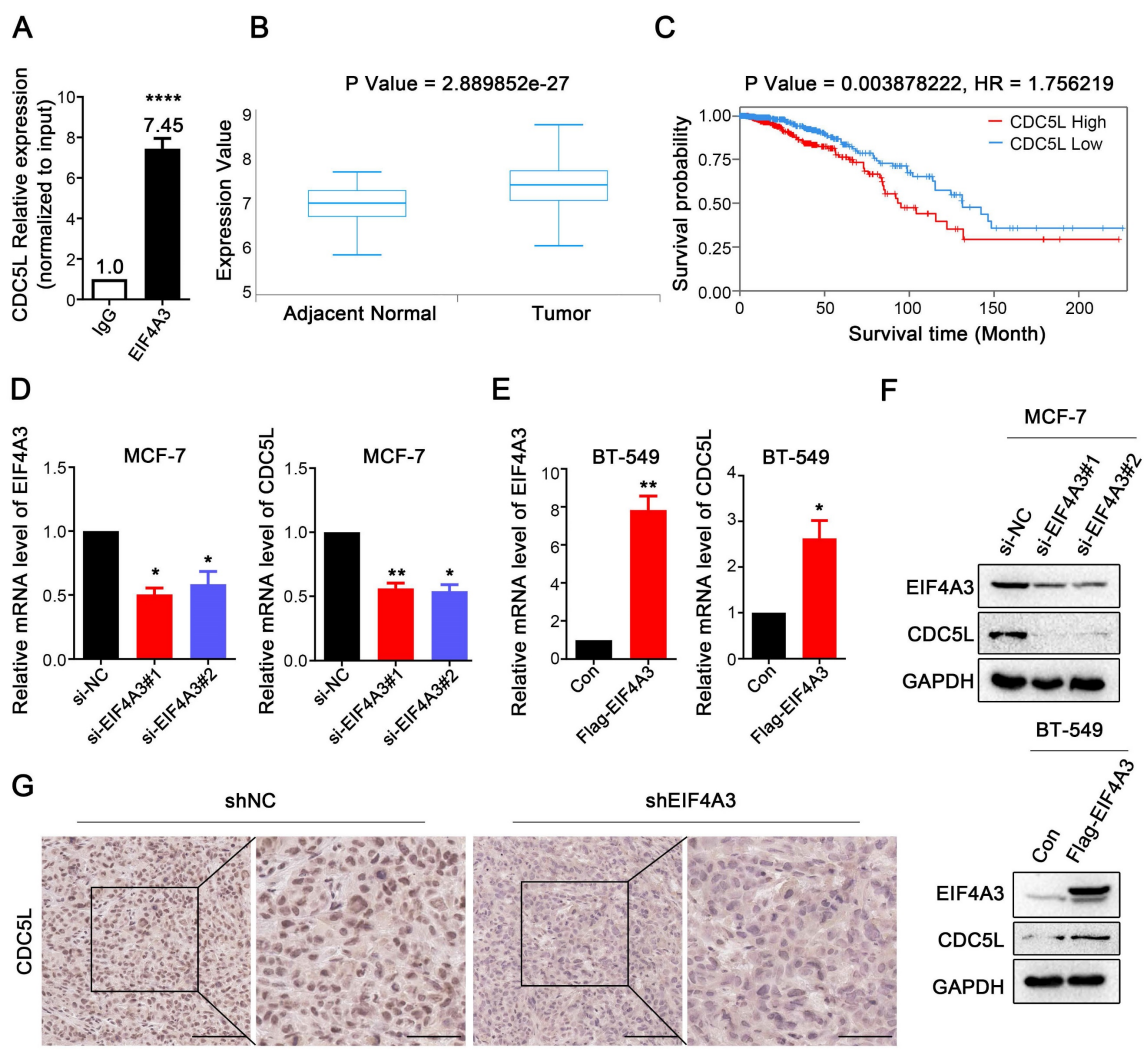
The expression of CDC5L was associated with EIF4A3. A: Interaction of EIF4A3 protein and CDC5L mRNA was detected by RIP and RT‒qPCR in MCF-7 cells (****P<0.0001); B: Expression of CDC5L mRNA in breast cancer and adjacent tissues in TCGA; C: Correlation between CDC5L mRNA expression level and overall survival (OS) of breast cancer patients in TCGA; D-F: Effect of abnormal expression of EIF4A3 on CDC5L at the transcription (D-E) and translation (F) levels by RT‒qPCR and Western blotting (*P<0.05; ** *P*<0.01); G: Analysis of CDC5L expression in xenograft tumors by immunohistochemistry (Scale bar: 100 μm on the left and 50 μm on the right).

To validate the conjugation between CDC5L mRNA and EIF4A3 protein, we used RIP-RT‒qPCR. The results showed that CDC5L was significantly enriched in the anti-EIF4A3 group, and the abundance was 7.45 times higher than that in the anti-IgG group (**Figure 6A**). Analysis of the TCGA database showed that CDC5L was significantly overexpressed in breast cancer tissues compared with adjacent tissues (**Figure 6B**), and patients with high CDC5L expression had poorer survival and prognosis (**Figure 6C**). The clinicopathological associations of EIF4A3 and CDC5L in human breast cancer of TCGA were provided in **Table S5**. There was a correlation between CDC5L expression and stage M. The higher the CDC5L expression, the less likely there was to be metastasis (P = 0.047). Next, we further detected the impact of abnormal EIF4A3 expression on CDC5L at the transcriptional and translational levels. The results showed that the expression of CDC5L was positively correlated with EIF4A3 expression at the transcriptional and translational levels (**Figure 6D-F**). Immunohistochemical staining was performed on previously collected tumors from the shNC and shEIF4A3 groups, and the staining of CDC5L was lighter and the expression was lower in the EIF4A3-depleted group than in the control group (**Figure 6G**). The above results indicated a positive correlation between CDC5L and EIF4A3.

### The EIF4A3-CDC5L axis promotes the proliferation of breast cancer cells

**Figure 7:**
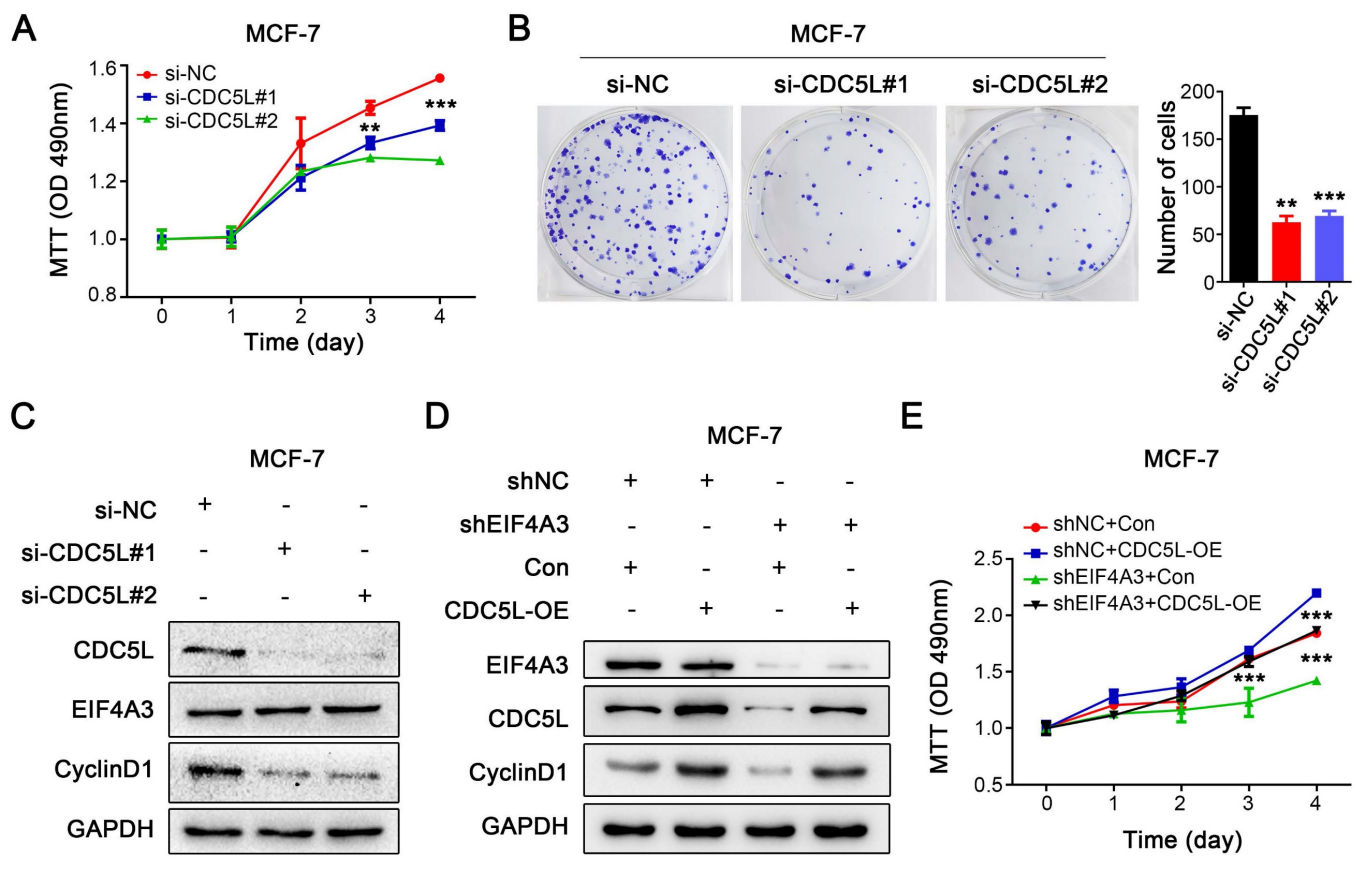
The proliferation of breast cancer cells was promoted via the EIF4A3-CDC5L axis. A-B: Effect of CDC5L knockdown on the proliferation of MCF-7 cells by MTT (A) and colony formation (B) (**P<0.01; ***P<0.001); C: Analysis of EIF4A3 and CyclinD1 expression after knockdown CDC5L in MCF-7 cells; D: The expression of EIF4A3, CDC5L and CyclinD1 was detected by Western blotting in a rescue experiment. E: Rescue experiment to analyze the effect of proliferation ability by MTT in MCF-7 cells (***P<0.001).

Because of the close relationship between CDC5L and the malignant process of breast cancer, we knocked down CDC5L in MCF-7 cells using siRNA. MTT assays and colony formation assays were performed to detect the effect on the proliferation of breast cancer cells. Knocking down the expression of CDC5L significantly inhibited the proliferation of MCF-7 cells (**Figure 7A-B**). After downregulation of CDC5L expression, the expression level of CyclinD1 protein decreased, but it did not affect the protein expression of EIF4A3 (**Figure 7C**). We then performed a rescue experiment to clarify the relationship. We observed that CDC5L overexpression elevated CyclinD1 expression with the knockdown of EIF4A3 first at the same time, which could be blocked by EIF4A3 silencing (**Figure 7D**). The proliferation ability of cells in the EIF4A3 knockdown group was significantly decreased compared to that in the control group. The proliferation ability of cells in the CDC5L overexpression group was significantly increased compared with that in the control group. The proliferation ability of the cells in the EIF4A3 knockdown group with CDC5L overexpression at the same time increased, which was almost the same as that of the control group (**Figure 7E**). These results suggested that EIF4A3 promoted the proliferation of human breast cancer cells by a CDC5L-related mechanism.

## Discussion

Breast cancer is the most common cancer and leading cause of cancer mortality in women worldwide^14^, ^15^. Despite recent advances in cancer therapy, there is still great room for progress in molecular targeted therapy of breast cancer. Abnormal cell proliferation has been observed in various tumors and plays a key role in the development of breast cancer.

Recently, systematic analysis of the biological role of EIF4A3 in human cancers suggested that it has the potential to be a diagnostic marker or therapeutic target in cancers^16^, ^17^. In this paper, we explored the potential effects of EIF4A3 on cell proliferation in breast cancer. The results showed that EIF4A3 was significantly upregulated in breast cancer and that high EIF4A3 expression correlated with poor prognosis in breast cancer patients. EIF4A3 expression was relatively higher in MCF-7 and T47D cells compared to the MCF10A, MDA-MB-231 and BT-549. Considering its expression pattern, EIF4A3 may be influenced by ER and/or PR. The Clinicopathological associations of EIF4A3 were analyzed in our breast cancer specimens. The clinical characteristics tumor site (left or right), cancer status (I-II or III), tumor size (≤ 2 cm or >2 cm), ER (negative or positive), PR (negative or positive), HER-2 (negative or positive) and triple (yes or no) were included. We noticed that there was no difference in the clinical features between EIF4A3 high- and low-expression groups. However, PR may regulate the expression of EIF4A3 because the p value was 0.0509. Further research was needed to explore this problem. Subsequently, further experiments revealed that knockdown of EIF4A3 significantly suppressed the proliferation and colony formation of breast cancer cells both *in vitro* and *in vivo*, accompanied by elevated levels of p21 and p27 and reduced expression of CyclinD1 and Survivin. This suggests that EIF4A3 possesses tumor-promoting activity in breast cancer.

As a core component of the EJC, EIF4A3 is an important RNA binding protein that plays pivotal roles in RNA metabolism, including mRNA splicing and trafficking and RNA surveillance^18^. EIF4A3 can be phosphorylated at threonine 163 (T163) by cyclin-dependent protein kinases 1 and 2 (CDK1, CDK2) in a cell cycle-dependent manner, participating in nonsense-mediated mRNA decay (NMD)^19^. As an RNA binding protein, EIF4A3 can directly bind RNA. Robust evidence suggests that EIF4A3 can affect the expression level of circRNA by participating in the back-splicing of circRNA^20^-^23^. These circRNAs promote the proliferation, migration, EMT, and radio sensitivity of cancer cells and facilitate tumor progression. Furthermore, lncRNA SNHG16 could bind to EIF4A3 and interact with it to form a complex, ultimately regulating the stability of RhoU mRNA^24^.

Given the RNA binding capacity of EIF4A3, we performed RIP-seq in this study to explore the RNAs that EIF4A3 binds to. We propose that as an effector of EIF4A3, CDC5L is regulated by EIF4A3 and that EIF4A3 can bind to its mRNA, which in turn affects the expression of downstream CyclinD1 and promotes the proliferation of breast cancer cells. From the catRAPID we predicted that EIF4A3-protein could bind to CDC5L by the 5705-5954 region of CDC5L-mRNA. CDC5L, a cell cycle regulator important for the G2-M transition, plays an important role in tumorigenesis and is the most likely candidate oncogene^25^-^27^. CDC5L is required for the S-phase cell-cycle checkpoint, and depletion of CDC5L results in a decrease in S-phase cells^28^, which is consistent with our research that S-phase cells decreased in the EIF4A3-depleted group by downregulating the expression of CDC5L. Another study explored the interaction of DEAD-box helicase 21 (DDX21) and CDC5L in colorectal cancer progression, in which the function of DDX21 is similar to that of EIF4A3 (alias: DEAD-box helicase 48)^29^. However, the specific molecular mechanism that how EIF4A3 regulates CDC5L remains unclear and needs further in-depth study. Despite its preliminary character, this study clearly indicates the relationship between EIF4A3 and CDC5L.

In recent years, a novel 1,4-diacylpiperazine-selective EIF4A3 inhibitor was discovered, which exhibits cellular NMD inhibitory activity^30^. A similar case is the development of a novel EIF4A3 inhibitor that targets the EJC^31^. These novel molecular probes present useful tools for further study of EIF4A3 in clinical treatment and make it possible for EIF4A3 to be a promising therapeutic target for breast cancer.

In summary, our study showed that EIF4A3 was highly expressed in breast cancer, and depletion of EIF4A3 inhibited the proliferation of breast cancer cells both *in vitro* and *in vivo*. We first proposed the idea that EIF4A3 can bind to the mRNA of CDC5L and regulate its expression. These findings strongly suggest that EIF4A3 can be a novel therapeutic target for breast cancer.

## Supplementary Material

Supplementary figures and tables 1-3, 5.

Supplementary table 4.
