# Supplementary material for: EIF4A3 Promotes Cell Proliferation via CDC5L Upregulation in Human Breast Cancer Cells
Source: J Cancer. 2025 Mar 3;16(6):1958–70. doi: 10.7150/jca.108895 (PMC11905404; doi:10.7150/jca.108895)
Supplement: Supplementary file 1 — Supplementary figures and tables 1-3, 5. [file jcav16p1958s1.pdf]

Additional files

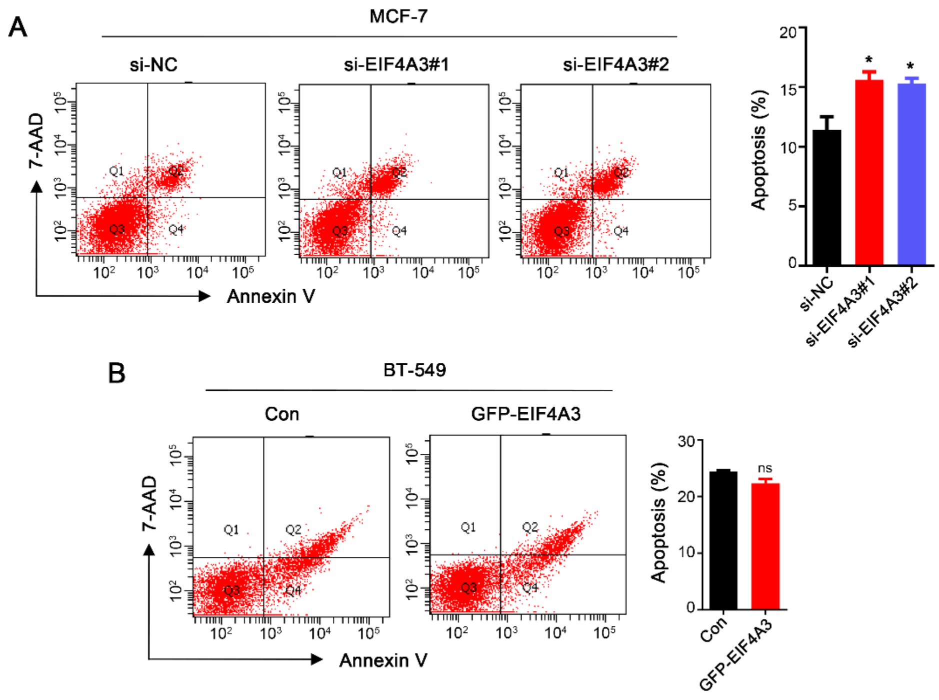

Figure S1 The effect of EIF4A3 on apoptosis of breast cancer cells.

A: Analysis of apoptosis after EIF4A3 knockdown in MCF-7 cells by flow cytometry (*P<0.05). B: Analysis of apoptosis after EIF4A3 overexpression in BT-549 cells by flow cytometry (ns: no significant).

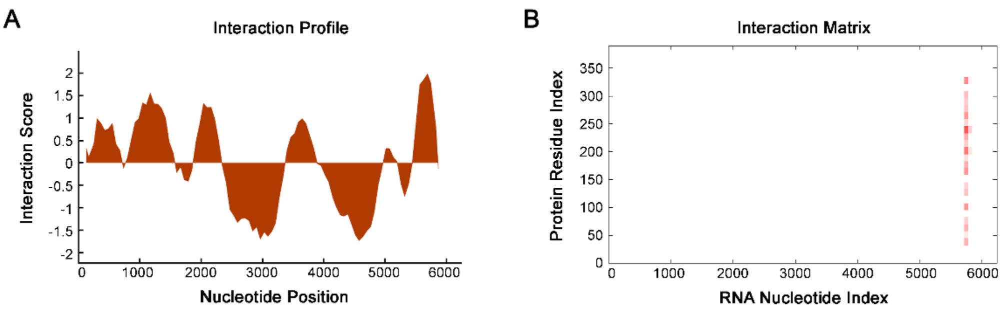

Figure S2 The interaction prediction of EIF4A3 and CDC5L.

A-B: The interaction profile (A) and the interaction matrix (B) of EIF4A3-protein and CDC5L-mRNA by using the catRAPID website.

Table S1 The sequence of siRNAs.

**ID**	**sense（5'-3'）**	**antisense（5'-3'）**
si-EIF4A3-homo-1	GCAAUCCAGCAACGAGCAATT	UUGCUCGUUGCUGGAUUGCTT
si- EIF4A3-homo-2	CCUCCAGUGUUUGGAUAUUTT	AAUAUCCAAACACUGGAGGTT
si- CDC5L-homo-1	GGAUGAACUUGAGAUGCUUTT	AAGCAUCUCAAGUUCAUCCTT
si- CDC5L-homo-2	GCGUGUAAAGGAAAUGAAATT	UUUCAUUUCCUUUACACGCTT

Table S2 The sequence of primer sets for real- time PCR assay.

**ID**	**Forward（5'-3'）**	**Reverse（5'-3'）**
EIF4A3	CCAGCAACGAGCAATCAAGC	CAGCCAACTCTCTTGTGGGA
CDC5L	AATCCCGAGGATGGAATGGC	ACGCTCTGCATCTCGTATGG
GAPDH	GTGGACCTGACCTGCCGTCT	GGAGGAGTGGGTGTCGCT

Table S3 Clinicopathological associations of EIF4A3 in human breast cancer

Clinicopathologic parameter		EIF4A3 (n=107)		P value
		low(%)	high(%)	
Tumor site	Left	10（9.34）	41（38.32）	0.4623
	Right	8（7.48）	48（44.86）	
Cancer status	I-II	17（15.89）	46（42.99）	0.2897
	III	8（7.48）	36（33.64）	
Tumor size(cm)	≤ 2cm	10（9.34）	42（39.25）	0.5173
	>2cm	8（7.48）	47（43.93）	
ER	Negative	16（14.95）	47（43.93）	0.5522
	Positive	9（8.41）	35（32.71）	
PR	Negative	20（18.69）	48（44.86）	0.0509
	Positive	5（4.67）	34（31.78）	
Her2	Negative	12（11.21）	40（37.39）	0.9455
	Positive	13（12.15）	42（39.25）	
Triple	Yes	7（6.54）	27（25.23）	0.6432
	No	18（16.82）	55（51.41）	

Table S4 The prediction of the bind of EIF4A3-protein and CDC5L-mRNA by using the catRAPID website.

The table contains the protein region, the RNA region, the raw score and the discriminative power (DP) of each interaction.

Table S5 Clinicopathological associations of EIF4A3 and CDC5L in human breast cancer of TCGA

Clinicopathologic parameter		EIF4A3 (n=918)		P value
		low(%)	high(%)	
Gender	Female	457 (49.78%)	450 (49.02%)	0.0639
	Male	2 (0.22%)	9 (0.98%)	
Age	> 58	213 (23.20%)	226 (24.62%)	0.4279
	≤ 58	246 (26.80%)	233 (25.38%)	
Pathologic stage	I-II	345 (37.58%)	353 (38.45%)	0.5884
	III-IV	114 (12.42%)	106 (11.55%)	
Stage T	T1-T2	390 (42.48%)	392 (42.70%)	0.926
	T3-T4	69 (7.52%)	67 (7.30%)	
Stage N	N0-N1	375 (40.85%)	384 (41.83%)	0.4854
	N2-N3	84 (9.15%)	75 (8.17%)	
Stage M	M0	451 (49.13%)	450 (49.02%)	>0.9999
	M1	8 (0.87%)	9 (0.98%)	

Clinicopathologic parameter		CDC5L (n=918)		P value
		low(%)	high(%)	
Gender	Female	454 (49.46%)	453 (49.35%)	>0.9999
	Male	5 (0.54%)	6 (0.65%)	
Age	> 58	221 (24.07%)	218 (23.75%)	0.8949
	≤ 58	238 (25.93%)	241 (26.25%)	
Pathologic stage	I-II	343 (37.36%)	355 (38.67%)	0.3951
	III-IV	116 (12.64%)	104 (11.33%)	
Stage T	T1-T2	388 (42.27%)	394 (42.92%)	0.6424
	T3-T4	71 (7.73%)	65 (7.08%)	
Stage N	N0-N1	373 (40.63%)	386 (42.05%)	0.2953
	N2-N3	86 (9.37%)	73 (7.95%)	
Stage M	M0	446 (48.58%)	455 (49.56%)	0.047*
	M1	13 (1.42%)	4 (0.44%)	

(*P<0.05)
